# Distinct clusters of stunted children in India: An observational study

**DOI:** 10.1111/mcn.12592

**Published:** 2018-02-23

**Authors:** Mark A. Green, Daniel J. Corsi, Ivan Mejía‐Guevara, S. V. Subramanian

**Affiliations:** ^1^ Department of Geography and Planning University of Liverpool Liverpool UK; ^2^ Ottawa Hospital Research Institute Ottawa Ontario Canada; ^3^ Centre for Population Health Sciences Stanford University Stanford California USA; ^4^ School of Public Health Harvard T.H. Chan Boston Massachusetts USA

**Keywords:** children, India, latent class analysis, socio‐economic factors, stunting, undernutrition

## Abstract

Childhood stunting is often conceptualised as a singular concept (i.e., stunted or not), and such an approach implies similarity in the experiences of children who are stunted. Furthermore, risk factors for stunting are often treated in isolation, and limited research has examined how multiple risk factors interact together. Our aim was to examine whether there are subgroups among stunted children, and if parental characteristics influence the likelihood of these subgroups among children. Children who were stunted were identified from the 2005–2006 Indian National Family Health Survey (*n* = 12,417). Latent class analysis was used to explore the existence of subgroups among stunted children by their social, demographic, and health characteristics. We examined whether parental characteristics predicted the likelihood of a child belonging to each latent class using a multinomial logit regression model. We found there to be 5 distinct groups of stunted children; “poor, older, and poor health‐related outcomes,” “poor, young, and poorest health‐related outcomes,” “poor with mixed health‐related outcomes,” “wealthy and good health‐related outcomes,” and “typical traits.” Both mother and father's educational attainment, body mass index, and height were important predictors of class membership. Our findings demonstrate evidence that there is heterogeneity of the risk factors and behaviours among children who are stunted. It suggests that stunting is not a singular concept; rather, there are multiple experiences represented by our “types” of stunting. Adopting a multidimensional approach to conceptualising stunting may be important for improving the design and targeting of interventions for managing stunting.

**Key messages**

- The determinants of stunting are numerous, complex, and interacting; however, current research fails to consider the multidimensional nature of stunting rather treating it as a singular concept.
- Our paper demonstrates the existence of five distinct types of stunted individuals in terms of health, social, and parental characteristics.
- Applying our multidimensional approach will help improve our understanding of the condition, as well as how to design effective interventions.

## 1 INTRODUCTION

Stunting is the chronic retardation of child growth as a result of nutritional inadequacies and defined by low height for age. The World Health Organisation (WHO) estimated that in 2016, there were 155 million children aged under 5 years (23.8%) globally who were stunted (WHO, 2017). Although India has seen large improvements towards tackling stunting with the estimated prevalence declining from 51% of all Indian children under 5 in 2005–2006 (Black et al., 2008) to 33% in 2015–2016 (Corsi, Meija, & Subramanian, 2016a), India still has the largest global burden of childhood undernutrition. Although there has been progress in tackling stunting both internationally and in India recently, aided by investment towards achieving Millennium Development Goal 1, India is unlikely to achieve the goal set by the United Nations (Ministry of Women and Child Development, 2014; United Nations, 2015) or future goals such as the Sustainable Development Goals (de Onis et al., 2013).

Stunting reflects the accumulation of micronutrient deficiencies typically as a result of undernutrition. A lack of specific nutrients can lead to stunted children having reduced immunological capacity to fight against (infectious) disease(s) (Black et al., 2008; Schaible & Kaufmann, 2007). Stunting itself can lead to longer term implications for child and subsequent adult health (UNICEF, 2013). It can also produce social implications as well. For example, children who are stunted are more likely to have poorer cognitive development that can restrict their educational achievement and future employment prospects (Crookston et al., 2011; Grantham‐McGregor et al., 2007; Martorell et al., 2010). The impact of stunting can be intergenerational with stunted mothers more likely to have premature children who then typically suffer from retarded growth (Grantham‐McGregor et al., 2007; Subramanian, Ackerson, & Davey Smith, 2010; UNICEF, 2013). The combination of the high prevalence of stunting alongside these associated health and social implications makes stunting an important policy consideration in India.

Current epidemiological and public health studies tend to focus on single risk factors of stunting rather than explore the complex interplay between multiple factors. Although there is growing evidence that childhood stunting is influenced by multifactorial drivers (Corsi, Meija, & Subramanian, 2016b; Danaei et al., 2016; Fenske, Burns, Hothorn, & Rehfuess, 2013), there have been no studies to our knowledge that have sought to explore the existence of heterogeneity among stunted children. Previous reviews on the effectiveness of nutritional interventions to prevent stunting have only reported limited success in reducing the prevalence of stunting (Bhutta et al., 2008, 2013; Dewey & Adu‐Afarwuah, 2008). One possible explanation for this is that policy interventions are often delivered to all children who are stunted together and who are effectively treated as a single homogenous entity. This approach may not be an efficient distribution of resources if individual characteristics (and how individuals may respond to an intervention) are not similar. Failure to incorporate multidimensional explanations for stunting characteristics will also miss out on understanding the wider determinants of stunting and, hence, limit our ability to design effective interventions.

Our study presents an alternative approach to exploring stunting in children. Using latent class analysis to explore similarities in multivariable associations across observations, we identify five “types” of stunted children in a representative survey of India (2005–2006). We also analyse the role of parental characteristics on latent class membership. Previous research has demonstrated the importance of parental characteristics such as education (Bhutta et al., 2013), body mass index (BMI) (Subramanian et al., 2010), height (Subramanian, Ackerson, Smith, & John, 2009), and whether the mother was married as a child (Raj et al., 2010) on the risk of their children being stunted (also see Corsi et al., 2016b; Danaei et al., 2016).

## 2 METHODS

### 2.1 Data

Data were taken from the 2005–2006 Indian National Family Health Survey (NFHS). The NFHS is the largest and most recent representative survey currently available that includes objective data on child anthropometry. We focused on children aged between 6 months and 5 years. We selected all singleton children whose mothers (the survey does not allow for proxy reporting, i.e., by other caregivers) had fully completed a 24‐hr dietary assessment (*n* = 32,360). Observations were excluded if children had missing data, or values of height and weight were implausible (defined as ±6 standard deviations WHO growth standards; WHO, 2006) resulting in a sample size of 28,984. Ethical approval was not required due to the study being secondary data analysis.

We identified whether children were stunted based on WHO guidelines. Height and weight measurements were converted into age‐ and sex‐specific *z*‐scores based on WHO child growth standards (WHO, 2006). Stunting was defined as any *z*‐score below −2 standard deviations. All children who were stunted were included in the analysis resulting in a final sample size of 12,417 (43%).

The selection of risk factors related to stunting, to be included as variables in our analysis, was based on the approach taken in a previous study (Corsi et al., 2016b). Each variable selected below was adapted from the UNICEF conceptual framework on the determinants of child undernutrition (Bhutta et al., 2008). We included variables to measure both social and health characteristics of children to account for different aspects of stunting. Most of the variables we used have standard definitions and have been described in more detail elsewhere (Barros et al., 2012; Corsi et al., 2016b). Variables included were

- Sex (male or female)
- Household wealth (split into quintiles)
- Life‐stage (categorised as 6 to 11 months, 12 to 23 months, 24 to 35 months, 36 to 47 months, and 48 to 59 months)
- Diet diversity based on a scoring system designed by Ruel and Menon (2002; categorised by quintile)
- Child was breastfed within 1 hr of birth or not
- Child had an infectious disease in previous 2 weeks or not
- Water source was through a piped connection to the dwelling or not
- Stools were safely disposed in the house or not
- Sanitation facility was improved (i.e., the hygienic separation of human excreta from contact with individuals using facilities such as a latrine flushing to a sewer or septic tank) or not
- House air quality (defined as use of nonsolid fuels, solid fuels in a separate kitchen, and solid fuels in a nonseparate kitchen) iodized salt used in household or not
- Child was fully vaccinated or not
- Vitamin A supplements taken or not

Seven variables of parental characteristics were also included as predictors of latent class membership. Mother's and father's education were included separately and divided into the following categories; no schooling, primary education, secondary education, and post‐secondary education. An issue with the inclusion of education is that household wealth is included in the latent class input variables, and education may be endogenous to household wealth. Although they are capturing slightly different concepts (i.e., material resources vs. parental cognition), the issue should be considered alongside the interpretation of our results. The height of the mother was also included and divided into the following categories (cm); ≥160.0, 155.0–159.9, 150.0–154.9, 145.0–149.9, and <145.0. For fathers' height, the categories were ≥170.0, 165.0–169.9, 160.0–164.9, 155.0–159.9, and <155.0. The height categories are different to reflect the different distribution of values between males and females (i.e., men were taller on average). Parental BMI was calculated separately by dividing body mass (kg) by height‐squared (m^2^) and further split into groups based on WHO defined cut offs; underweight (<18.5), normal (18.5–24.9), and overweight (≥25). Finally, we included whether the mother was married before the age of 18 years.

### 2.2 Statistical analysis

Latent class analysis (LCA) was used to explore the existence of homogenous groups within children who are stunted. LCA is a finite mixture model that seeks to identify a latent structure within data through a probabilistic model (Collins & Lanza, 2010). The aim is to identify a categorical latent variable that is not directly measured but captured through other observed variables. Groups are identified based on the multidimensional distribution of variables.

As LCA is an exploratory method, a decision must be made on the number of latent classes that best describes the underlying structure of the data. To identify the most appropriate number of classes, we ran several models for a range of solutions between 2 and 10. We did not consider a larger number of latent classes because we wanted the chosen model to be parsimonious. Model fit was assessed using the adjusted Bayesian Information Criterion, consistent Akaike Information Criterion, and G‐squared statistic (Collins & Lanza, 2010).

One strength of LCA is that covariates can be included in the model to predict how factors are associated with class membership (Collins & Lanza, 2010). Covariates were modelled using a multinomial logit model. Mother's and father's education level, BMI category, and height were each included as covariates, as well as whether the mother was married under the age of 18. We report odds ratios for the model and the 95% confidence intervals for these estimates.

All analyses were undertaken using SAS v9.3 and the PROC LCA procedure (Lanza et al., 2007). Sample weights were included in the analysis allowing our observations to be representative and to account for the survey design (although PROC LCA cannot account for the stratified multistage cluster design for how the data were collected, limiting the representativeness of the analyses).

## 3 RESULTS

Table 1 presents summary statistics of the characteristics of children who were stunted and for the whole sample to help contextualise the data. There were slightly more males compared with females in our sample of stunted children, although this only differed slightly from all children. There was a greater proportion of stunted children from poorer households compared with the whole sample. Stunted children were on average older than the average for the entire sample. Stunted children had less diverse diets and lower prevalence of some health‐related measures (e.g., vaccinations, safe disposal of stools) in comparison with the whole sample. The characteristics of parents also followed these patterns.

**Table 1:** Sample characteristics of stunted children (sample weights were applied)

		Stunted children (percentage)	All children (percentage)
Panel A: Latent class analysis input variables			
Males		51.9	51.6
Females		48.1	48.5
Household wealth quintile	1 (lowest)	34.7	28.6
	2	24.9	21.6
	3	19.1	18.9
	4	14.9	17.6
	5 (highest)	6.9	13.4
Life stage	6–11 months	8.7	14.9
	12–23 months	30.2	29.2
	24–35 months	30.7	28.1
	36–47 months	14.9	11.8
	48–59 months	15.5	9.3
Diet diversity score quintile	1 (lowest)	35.6	31.4
	2	25.5	25.3
	3	21.6	22.3
	4	10.8	11.8
	5 (highest)	6.6	9.3
Breast fed within 1 hr of birth		28.1	23.4
Infectious disease in past 2 weeks		12.9	28.5
Water drinking source was through a piped connection		11.6	17.8
Safe disposal of stools		11.6	15.5
Improved sanitary facility		15.5	22.4
Household air quality	Nonsolid fuels	11.9	18.3
	Solid fuels in separate kitchen	52.8	51.1
	Solid fuels in nonseparate kitchen	35.3	30.6
Iodized salt used		41.9	46.2
Fully vaccinated		37.3	40.9
Vitamin A supplement taken		18.5	20.1
Panel B: Covariates for explaining class membership			
Mothers education	No schooling	59.3	50.0
	Primary	14.1	14.0
	Secondary	22.2	26.6
	Post‐secondary	4.5	9.4
Mothers body mass index	Underweight	44.8	41.1
	Normal	51.5	53.1
	Overweight	3.7	5.8
Mothers height (cm)	<145	15.8	12.1
	145–149.99	31.6	27.0
	150–154.99	32.4	33.5
	155–159.99	15.8	19.8
	≥160	4.5	7.6
Mother was married before 18		66.8	60.7
Fathers education	No schooling	34.6	29.3
	Primary	16.8	15.2
	Secondary	36.4	37.6
	Post‐secondary	12.2	17.9
Fathers body mass index	Underweight	36.2	31.4
	Normal	58.0	59.5
	Overweight	5.8	9.1
Fathers height (cm)	<155	9.0	7.0
	155–159.99	20.8	17.5
	160–164.99	31.5	29.9
	165–169.99	24.1	26.5
	≥170	14.6	19.1

**Figure 1:**
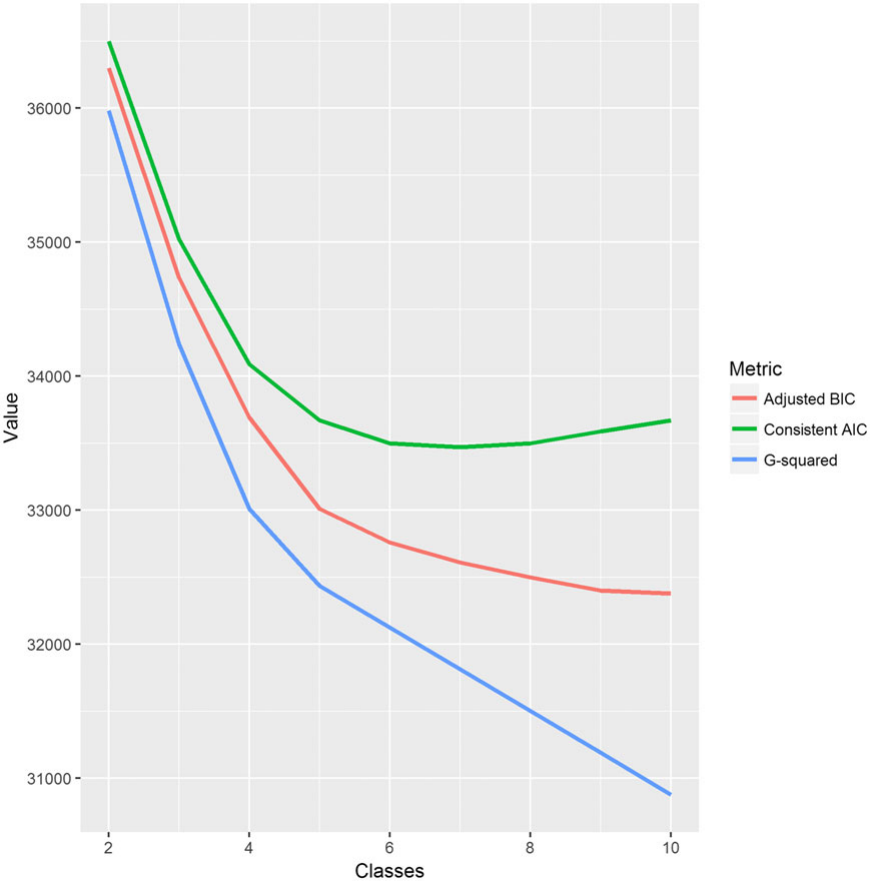
Examining model fit statistics by the number of latent classes. AIC = Akaike Information Criterion; BIC = Bayesian Information Criterion

Figure 1 presents the analysis of the number of latent classes that best captures the underlying structure of the data. Although an increasing number of groups typically resulted in an improved model across each measure of model fit, the rate of these improvements decreased with increasing number of solutions. Both the consistent Akaike Information Criterion and adjusted Bayesian Information Criterion have a clear “knee point” at five classes whereby increasing number of classes adds little additional information. The G‐squared also has a kink at five but continues decreasing. However, the measure is less useful with large samples (Collins & Lanza, 2010). We selected a five class solution for our analysis.

**Figure 2:**
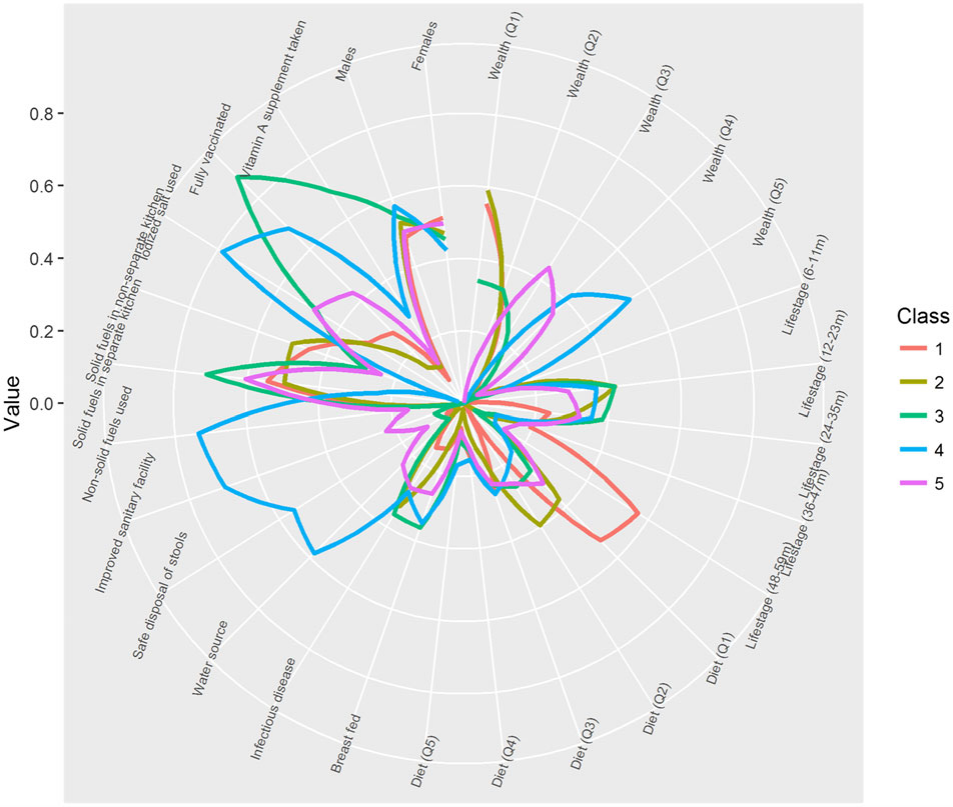
A radial plot of the conditional probabilities for each latent class

Table 2 presents the conditional probabilities of each latent class and the latent class prevalences (Figure 2 presents the conditional probabilities using a radial plot to aid interpretation). We named each latent class and described their characteristics below:

1. Poor, older, and poor health‐related outcomes: Although the characteristics are mostly similar to “poor, young and poorest health‐related outcomes,” there are some key differences. The children mainly differ based on life stage being older. The latent class also have a higher prevalence of children fully vaccinated, fewer children suffering from infectious diseases, and a bimodal distribution for the diet variable.
2. Poor, young, and poorest health‐related outcomes: The latent class contained the largest probability of the lowest two quintiles of household wealth. They are also the youngest class compared with the other classes. Characteristics were largely the worst in comparison with the other classes. Diet diversity was low, and although “poor, older and poor health‐related outcomes” had the largest probability for Quintile 1, the class has the largest combined probability for Quintiles 1 and 2. The class also had some of the poorest health‐related characteristics especially for hygiene and sanitation, household air quality, and full vaccinations. It is the largest class.
3. Poor with mixed health‐related outcomes: There are higher probabilities of children in the lower quintiles of household wealth. The class displays relatively good health‐related characteristics, with the highest probability for Vitamin A supplements, full vaccinations, and breastfeeding. However, it also has the highest probability for infectious disease and low probabilities for the hygiene, sanitation, and household air quality variables. The majority were aged between 1 and 3 years old. It is the second smallest class as well.
4. Wealthy and good health‐related outcomes: Most children in the class are in the top two quintiles of household wealth. They displayed the best health‐related outcomes characteristics in comparison with the other clusters. However, they did not perform the best for every health‐related outcome variable, for example, fully vaccinated or Vitamin A supplements. The majority were aged between 1 and 3 years old. It was the smallest class.
5. Typical traits: The conditional probabilities largely fall in the middle in comparison with the other latent classes. There are few features that make the class distinctive other than this.

**Table 2:** The characteristics of a five‐group latent class analysis of stunted children

		Class 1	Class 2	Class 3	Class 4	Class 5
Prevalence (*ϒ*)		0.190	0.349	0.103	0.107	0.251
Conditional probabilities (*ρ*)						
Males		0.485	0.527	0.545	0.576	0.501
Females		0.515	0.473	0.455	0.424	0.499
Household wealth quintile	1 (lowest)	0.554	0.591	0.340	0.000	0.001
	2	0.321	0.309	0.331	0.001	0.167
	3	0.106	0.092	0.229	0.040	0.442
	4	0.019	0.009	0.099	0.420	0.347
	5 (highest)	0.001	0.000	0.001	0.539	0.043
Life stage (months)	6–11	0.000	0.139	0.065	0.081	0.091
	12–23	0.002	0.420	0.419	0.368	0.291
	24–35	0.237	0.296	0.385	0.355	0.322
	36–47	0.194	0.146	0.094	0.095	0.166
	48–59	0.567	0.000	0.036	0.101	0.131
Diet diversity score quintile	1 (lowest)	0.533	0.372	0.261	0.186	0.310
	2	0.000	0.396	0.270	0.223	0.259
	3	0.255	0.160	0.237	0.264	0.235
	4	0.145	0.057	0.129	0.155	0.120
	5 (highest)	0.067	0.015	0.103	0.171	0.076
Breast fed within 1 hr of birth		0.132	0.118	0.362	0.348	0.262
Infectious disease in past 2 weeks		0.143	0.333	0.360	0.287	0.275
Water drinking source was through a piped connection		0.026	0.019	0.057	0.583	0.237
Safe disposal of stools		0.056	0.028	0.070	0.551	0.117
Improved sanitary facility		0.036	0.023	0.085	0.696	0.227
Household air quality	Nonsolid fuels	0.005	0.003	0.000	0.735	0.154
	Solid fuels in separate kitchen	0.544	0.497	0.713	0.247	0.604
	Solid fuels in nonseparate kitchen	0.451	0.500	0.286	0.018	0.242
Iodized salt used		0.312	0.300	0.478	0.785	0.486
Fully vaccinated		0.275	0.139	0.882	0.682	0.432
Vitamin A supplement taken		0.077	0.118	0.690	0.283	0.131

There was little variation in proportion of males and females in each latent class suggesting that our latent classes are largely independent of sex.

Table 3 presents the results exploring the association between parental characteristics and latent class membership. The group “typical traits” (Class 5) were selected as the comparator class for interpreting the estimates. Mothers who were married before the age of 18, lower educational attainment, and underweight parents were positively associated with membership of Classes 1 to 3 compared with “typical traits.” Relationships were fairly consistent between mothers and fathers, other than for “poor with mixed health‐related outcomes” (Class 3) where mother's education was not important. The direction of these associations to “wealthy and good health‐related outcomes” (Class 4) was opposite (although there was little association for underweight fathers).

**Table 3:** A multinomial logit model of the association between characteristics of parents and class membership

		Class 1	Class 2	Class 3	Class 4	Class 5 (reference)
Mothers education	No schooling	2.99 [2.19, 4.09]	3.02 [2.27, 4.03]	0.67 [0.37, 1.22]	0.56 [0.33, 0.95]	
	Primary	Reference				
	Secondary	0.35 [0.23, 0.54]	0.32 [0.22, 0.48]	0.72 [0.36, 1.46]	3.12 [2.13, 4.56]	
	Post‐secondary	0.17 [0.03, 0.87]	0.11 [0.03, 0.47]	0.44 [0.08, 2.42]	10.29 [6.33, 16.71]	
Mothers body mass index	Underweight	1.32 [1.08, 1.61]	1.59 [1.34, 1.90]	1.75 [1.23, 2.50]	0.73 [0.58, 0.93]	
	Normal	Reference				
	Overweight	0.53 [0.28, 1.01]	0.38 [0.21, 0.69]	0.46 [0.12, 0.169]	3.40 [2.30, 5.02]	
Mothers height (cm)	<145	1.07 [0.78, 1.48]	1.25 [0.94, 1.66]	1.16 [0.70, 1.91]	1.41 [0.98, 2.02]	
	145–149.99	1.05 [0.83, 1.34]	1.02 [0.83, 1.27]	1.33 [0.91, 1.94]	0.83 [0.63, 1.09]	
	150–154.99	Reference				
	155–159.99	0.98 [0.73, 1.31]	0.84 [0.65, 1.09]	0.82 [0.53, 1.25]	1.60 [1.19, 2.33]	
	≥160	0.70 [0.45, 1.08]	0.80 [0.54, 1.19]	0.78 [0.38, 1.58]	1.50 [0.96, 1.33]	
Mother was married before 18		1.56 [1.25, 1.95]	1.72 [1.40, 2.11]	1.16 [0.83, 1.63]	0.52 [0.42, 0.65]	
Fathers education	No schooling	3.02 [2.26, 4.03]	3.14 [2.41, 4.11]	1.31 [0.77, 2.22]	0.51 [0.27, 0.98]	
	Primary	Reference				
	Secondary	0.69 [0.52, 0.91]	0.68 [0.53, 0.86]	0.48 [0.33, 0.70]	1.82 [1.23, 2.71]	
	Post‐secondary	0.58 [0.35, 0.94]	0.44 [0.27, 0.70]	0.34 [0.17, 0.70]	2.32 [1.46, 3.68]	
Fathers body mass index	Underweight	1.84 [1.36, 2.49]	1.62 [1.21, 2.16]	1.47 [0.88, 2.47]	0.96 [0.65, 1.41]	
	Normal	Reference				
	Overweight	0.30 [0.11, 0.83]	0.30 [0.12, 2.16]	0.25 [0.04, 1.50]	4.72 [2.89, 7.70]	
Fathers height (cm)	<155	1.25 [0.73, 2.15]	1.19 [0.74, 1.92]	1.17 [0.56, 2.47]	0.18 [0.06, 0.54]	
	155–159.99	0.90 [0.63, 1.28]	0.91 [0.67, 1.24]	0.64 [0.36, 1.14]	0.67 [0.44, 1.03]	
	160–164.99	Reference				
	165–169.99	0.83 [0.60, 1.14]	0.66 [0.48, 0.89]	0.70 [0.43, 1.14]	1.03 [0.74, 1.43]	
	≥170	0.65 [0.42, 1.14]	0.61 [0.41, 0.93]	0.55 [0.26, 1.19]	0.65 [0.44, 0.98]	

*Note*. We present odds ratios with 95% confidence intervals in brackets.

Height displayed less certainty in the direction of estimates for both mothers and fathers with most confidence intervals crossing a value of 1. Although taller mothers were positively associated with class membership of “wealthy and good health‐related outcomes” (Class 4), the result for father's height was contrary. Taller fathers were negatively associated with membership of “poor, young and poorest health‐related outcomes” (Class 2) compared with “typical traits” (Class 5).

## 4 DISCUSSION

Our study revealed five distinct “types” of stunted children in India demonstrating clear heterogeneity of stunting. An important contribution of our study is the examination of multiple risk factors and determinants within a mutually adjusted casual framework. A major limitation of many high profile studies in this field is the continued examination of single risk factors in isolation leading to biased effect estimates (Danaei et al., 2016). The high prevalence of stunting in India (and globally), combined with the associated health and social implications of stunting (Black et al., 2008; Schaible & Kaufmann, 2007; UNICEF, 2013), has seen considerable interest in how to effectively design interventions to tackle stunting (Bhutta et al., 2008, 2013). Treating childhood stunting as a singular concept may lead to a false dichotomy of the determinants and experiences of stunting, limiting our understanding of the issue and our ability to tackle it.

The multidimensional nature of childhood stunting identified in our study has important implications for future policy. The distinct characteristics of each latent class suggest that differing strategies are required to tackle the issue. Ignoring the complex combination of characteristics that constitute each class may restrict the effectiveness of policies or lead to inefficient targeting of resources (Dewey & Adu‐Afarwuah, 2008; Fenske et al., 2013). For example, improving vaccination uptake among stunted children to protect against infectious diseases would appear important given that only 37.3% of stunted children were fully vaccinated. However, the policy would be less appropriate for “poor with mixed health‐related outcomes” (Class 3) who had a high prevalence of vaccination, where it may be better to focus on other issues such as improving access to clean water. Future research should explore the application of our latent classes within different interventions to assess how useful they are for delivering policy more efficiently.

The discovery of a “poor but mixed health‐related outcomes” cluster was a particularly important finding because it demonstrates the possibility to achieve good health‐related outcomes despite the imposing forces of a lack of wealth. Children in the class had the highest prevalence of children fully vaccinated and Vitamin A supplements taken but low prevalence of improved drinking water source or safe sanitation. The finding may suggest that it is useful when implementing interventions aimed at improving health‐related outcomes in poor and stunted children to improve education and awareness about access to health services as these represent potential successes. Wealth and neighbourhood conditions are less modifiable and, therefore, harder to address (Bhutta et al., 2008; van de Poel, Hosseinpoor, Speybroeck, Ourti, & Vega, 2008). However, the finding may simply relate to the successful delivery of programmes targeting low socio‐economic status areas or regions (e.g., to increase vaccinations).

The associations found with the covariates (as well as the relative importance of household wealth in determining the latent classes) are indicative of the importance of the social gradient in understanding latent classes. Although most stunting‐related interventions are aimed at improving nutrition (Bhutta et al., 2013), it is clear that for any intervention to be successful, they must be combined with the wider social context (Corsi et al., 2016b; Dewey & Adu‐Afarwuah, 2008). Tackling the social gradient will need to be more targeted than simply encouraging economic growth, because economic growth alone is not associated with improved child nutrition (Subramanyam, Kawachi, Berkman, & Subramanian, 2011). Although wealthy children have been previously shown to be less likely to be stunted (Corsi et al., 2016b; Fenske et al., 2013; van de Poel et al., 2008), our findings indicate that they make up their own latent class with more favourable health‐related outcomes compared with the other groups. They were, however, a small class, but nonetheless, it underscores that certain “better off” children may be at risk for stunting despite higher socio‐economic status.

Parental BMI was associated with class membership, although more consistently for mothers compared with fathers. This association may partly reflect the social gradient as well, because poorer individuals have been previously demonstrated to be more likely to be underweight (Bhutta et al., 2008; Subramanian, Corsi, Subramanyam, & Smith, 2013). However, there may also be an independent association. It is plausible that underweight parents pass on similar habits to the children. Previous research has shown that stunting displays an intergenerational aspect (Grantham‐McGregor et al., 2007; Subramanian et al., 2009; UNICEF, 2013). A similar interpretation may be derived for height as well, although the results were inconsistent. Our finding that fathers characteristics explain the type of stunting supports calls to move away from a “maternal” to a “household” understanding of the determinants of stunting (Corsi et al., 2016a).

There are several limitations to our approach. The data were cross‐sectional, and therefore, our ability to draw inferences about our groupings is limited. It will be important to replicate the approach using longitudinal observations to explore how the groups develop and change over time. We only included seven predictors of class membership, and it will be important to examine the association of additional factors that have been shown to be related to stunting such as the socio‐economic context of local regions (Subramanyam et al., 2011). LCA can only make observations about how factors are co‐associated within stunted children, and it does not show the strength of an association to the risk of stunting. The data we used were collected in 2005–2006 and are therefore outdated. They are, however, the most recent data available at the time of analysis. Irrespective of the date, it does not change the notion that the strength of the paper is its conceptual approach of exploring a typology of stunting. The NFHS is currently processing the data collected in the fourth survey wave (2015–2016), and it will be important to update our study using these newer data when they are released to examine how the situation has changed. The risk factor data are self‐reported and therefore potentially subject to bias. There were no alternative large representative data sets that included objective measures limiting the quality of our observations. The data used for the characteristics of fathers correspond to the mother's partner at the time of the survey. Although the majority of partners are the biological father of the child, it may introduce a small amount of bias in estimates. However, this should not distract from the importance of how adults within the household may influence experiences.

In conclusion, the causes of childhood stunting are complex and multidimensional. Our paper contributes to a literature that has largely examined stunting as a singular concept, demonstrating heterogeneity among stunted children. We hope that the approach outlined in this paper will help policy makers in designing effective interventions as opposed to more simplistic approaches that do not differentiate in terms of the individuals they target.

## CONFLICTS OF INTEREST

The authors declare that they have no conflicts of interest.

## CONTRIBUTIONS

SVS conceived the idea for the study. DC and IMG were involved in data preparation. MAG designed and undertook the analyses. All authors were involved in reviewing the analyses and in writing the manuscript.
